# Synthesis and Characterization of Zintl-Phase BaCd_2_P_2_ Quantum Dots for Optoelectronic Applications

**DOI:** 10.1021/acsnano.5c02271

**Published:** 2025-03-24

**Authors:** Matthew P. Hautzinger, Shaham Quadir, Benjamin Feingold, Reilly Seban, Arianna J. Thornton, Nikita S. Dutta, Andrew G. Norman, Ian A. Leahy, Muhammad Rubaiat Hasan, Kirill A. Kovnir, Obadiah G. Reid, Bryon W. Larson, Joseph M. Luther, Matthew C. Beard, Sage R. Bauers

**Affiliations:** † National Renewable Energy Laboratory, Golden, Colorado 80401, United States; ‡ Department of Chemistry, University of Colorado, Boulder, Colorado 80401, United States; § Department of Physics, Colorado School of Mines, Golden, Colorado 80401, United States; ∥ Department of Chemistry, Iowa State University, Ames, Iowa 50011, United States; ⊥ Ames National Laboratory, U.S. Department of Energy, Ames, Iowa 50011, United States; # Renewable and Sustainable Energy Institute, University of Colorado Boulder, Boulder, Colorado 80309, United States

**Keywords:** Zintl phases, quantum dots, colloidal, optoelectronics, superlattices, cation exchange

## Abstract

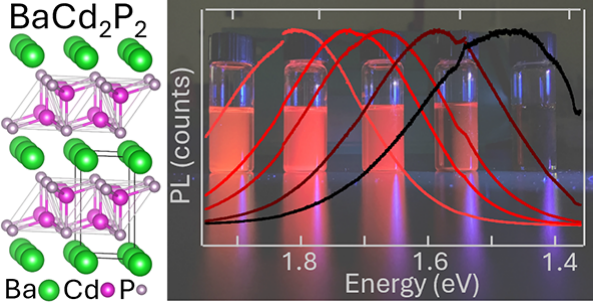

We demonstrate the growth of size-controlled, high optical quality Zintl-phase BaCd_2_P_2_ colloidal quantum dots (QDs), an emerging semiconductor absorbing/emitting in the red and predicted to have favorable defect chemistry. The QDs are grown via hot injection of a phosphorus precursor into a solution of solubilized Ba and Cd precursors. The absorbance and photoluminescence (PL) are tunable via growth temperature and show a bandgap ranging from 1.47 to 1.81 eV, depending on the size, which ranges from 3 to 9 nm based on electron microscopy. Selected area electron diffraction is used to determine that the BaCd_2_P_2_ QDs crystallize in the *P*3̅*m*1 space group, same as the bulk material. Raman spectroscopy, powder X-ray diffraction, and X-ray fluorescence studies further confirm that BaCd_2_P_2_ QDs match those of the crystalline phase bulk material. The high optoelectronic quality is assessed by quantification of long-lived photoexcited carriers (∼160 ns average weighting), as determined by time-resolved PL spectroscopy, and bright red visible emission (∼21% PL quantum yield) despite no complex surface passivation. Furthermore, a demonstration of thin-film fabrication is shown via a solid state ligand exchange protocol. This synthetic protocol enables researchers to explore and utilize BaCd_2_P_2_ Zintl-phase QDs, as well as adjacent compositions, for a variety of optoelectronic applications enabled by their semiconducting properties.

Recently, the Zintl-phase BaCd_2_P_2_ has been identified as an underexplored semiconductor with a 1.45 eV band gap, large optical absorption coefficient, and an absence of deep native point defects making it well suited for common optoelectronic applications including lighting, bioimaging, communication, and solar energy harvesting.^1^ Zintl-phase materials can be described as highly covalent polyanionic MX^*n*–^ (M = p-block or transition metal; X = electronegative elements: P, As, etc.) species interceded by highly electropositive cations A^*n*+^ (A = alkali or alkaline earths).^2^ While several theoretical and experimental studies hinted that AM_2_X_2_ compounds are promising optoelectronic semiconductors,^3−7^ it was not until the report on BaCd_2_P_2_,^1^ and very shortly after on CaZn_2_P_2_,^3^ that potentially high optoelectronic performance was demonstrated through measurements of bright band-edge photoluminescence (PL) and ∼30 ns carrier lifetimes, despite the materials being relatively unoptimized.

In BaCd_2_P_2_, CaZn_2_P_2_, and likely the other AM_2_X_2_ pnictides, the intrinsic vacancy and substitutional defects were shown via density functional theory calculations to either have extremely high formation enthalpies at relevant chemical potentials or be shallow enough electronically to not trap carriers (*i.e*., close to either the valence band maximum or conduction band minimum).^1,3^ The high optoelectronic quality measured from BaCd_2_P_2_ (synthesized from solid state reaction) and CaZn_2_P_2_ (deposited by reactive sputtering) is a strong indication that the calculated shallow electronic defects, which should not impact the photocarriers, has empirical merit even beyond intrinsic point defects. This is because these fabrication methods are well-known to result in emission-killing unpassivated surfaces and grain boundaries that are not considered by the calculations, as would be the case for III-Vs or group IV semiconductors grown via similar methods. BaCd_2_P_2_ powders also exhibited excellent stability in a wide range of harsh conditions (strongly basic environment, high temperature, etc.), altogether making a compelling case for wide usability in a variety of optoelectronic applications. Unfortunately, the currently available methods for preparing AM_2_X_2_ phosphides must be expanded, as they are either completely intractable or require significant growth engineering to be incorporated into optoelectronic platforms.

These observations about the interesting defect chemistry and the lack of existing synthetic routes of Zintl-phase materials inspired us to pursue the solution growth of colloidal Zintl-phase quantum dots (QDs), not routinely explored in the literature.^8^ QDs often suffer from undercoordinated surface states which can detrimentally impact the optoelectronic properties, particularly in pnictides.^9^ Typically, this is mitigated through designed surface passivation, yet exploring materials with shallow defect energies can lead to facile growth of high quality semiconducting QDs. In addition, QDs that do not require complex surface passivation (*i.e*. shells) are better suited for select applications where carrier diffusion length is of importance, which can be impeded in core@shell structures. Looking for synthetic inspiration to draw upon, colloidal InP,^10^ InAs,^11^ Cd_3_P_2_,^12^ and Cd_3_As_2_^13^ have all been grown as colloidal quantum dots with highly reactive tris(trimethylsilyl)-pnictogen precursors, which we aimed to adapt while exploring the growth parameters of these ternary QDs.

Here we show the synthesis of BaCd_2_P_2_ QDs as a demonstration of the capability to grow a general class of colloidal Zintl-phase QDs. The QDs have tunable absorbance/PL energies of 1.47–1.81 eV, with bright room-temperature PL without designed/additional surface passivation. Electron microscopy shows particle sizes of 3–9 nm, and a variety of characterization techniques are used to demonstrate BaCd_2_P_2_ QDs match the bulk phase. The carriers are moderately long-lived (up to 160 ns weighted average) as determined with time-resolved photoluminescence (TRPL) and the photoluminescence quantum yield (PLQY) was determined to be 21%. To provide a basis for incorporation into optoelectronic platforms, we demonstrate a solid-state ligand exchange procedure to deposit compact thin films. Thin-film quality was characterized with time-resolved microwave conductivity (TRMC). In addition, we discuss the spontaneous formation of BaCd_2_P_2_ QD superlattices, which typically require a reasonably tight size dispersion and strongly bound surface ligands to form. To demonstrate compositional control and possibilities of related compositions, a cation exchange reaction is achieved via ZnI_2_ treatment forming Ba(Cd_1–*x*_Zn_*x*_)_2_P_2_ QDs as indicated by optical spectroscopies.

## Growth of BaCd_2_P_2_ QDs via Hot Injection

To synthesize BaCd_2_P_2_ QDs, we adapted hot injection procedures used previously for pnictide materials.^10−14^ Mixtures of BaI_2_·2H_2_O, CdO, oleic acid (OA), trioctylphosphine oxide (TOPO), and octadecene (ODE) were dried under a vacuum on a Schlenk line. The mixture was then heated to 220 °C under N_2_ and both powders of Ba and Cd precursor were dissolved, with the BaI_2_·2H_2_O (presumably dried at this stage to BaI_2_) precursor taking longer than the CdO. The CdO is converted to Cd(oleate)_2_ at this temperature, which generates H_2_O as a byproduct. The solution was then cooled to 120 °C and held under vacuum to ensure removal of this byproduct and any other excess H_2_O. Failure to adequately dry the solution resulted in no growth of the QDs during the later steps.^15^ Further notes about the solubility of precursors are in the Supporting Information (SI), section 2.

After the Ba and Cd precursors were formed, the solution was brought to a specified reaction temperature (110–190 °C) and a mixture of stoichiometric tris(trimethylsilyl)phosphine ((TMSi)_3_P, 1:1 P:Cd) diluted in dry octadecene was injected. Please see the note about the safe handling of (TMSi)_3_P in Materials and Methods. After injection there was an instantaneous color change to red and then black during the growth, with the speed of this color change depending on the temperature (approximately 5 s (190 °C) to 1 min (110 °C) to turn red to black). The reaction mixture was allowed to react for specified times and cooled to room temperature by removing from heat with no intentional quenching. Further discussion exploring the growth conditions including ligand choice and phosphorus ratio are in the SI, section 2.

A simple proposed reaction is shown below:
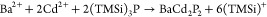
with oleate, TOPO, iodide, and trimethylsilyl groups as possible ligands terminating the surface. As noted in SI section 2, TOPO can be substituted for oleylamine (OLAM).

The QDs were then washed and centrifuged to remove the ODE and unreacted precursors, first with methyl acetate (MeOAc) for one washing round and then acetone for two more rounds. This procedure was determined by attempts with various antisolvents. When MeOAc is used exclusively as an antisolvent (*i.e*. three rounds of MeOAc precipitation and centrifuging), the QDs lose their colloidal stability, suggesting the ligands are removed from the surface of the QDs. When exclusively acetone, ethanol, or acetonitrile was used as the washing solvent, unreacted precursor from the growth was not effectively removed and would precipitate slowly from the QD solutions as a white gel-like species over time.

## Structural Characterization of BaCd_2_P_2_ QDs

**Figure 1:**
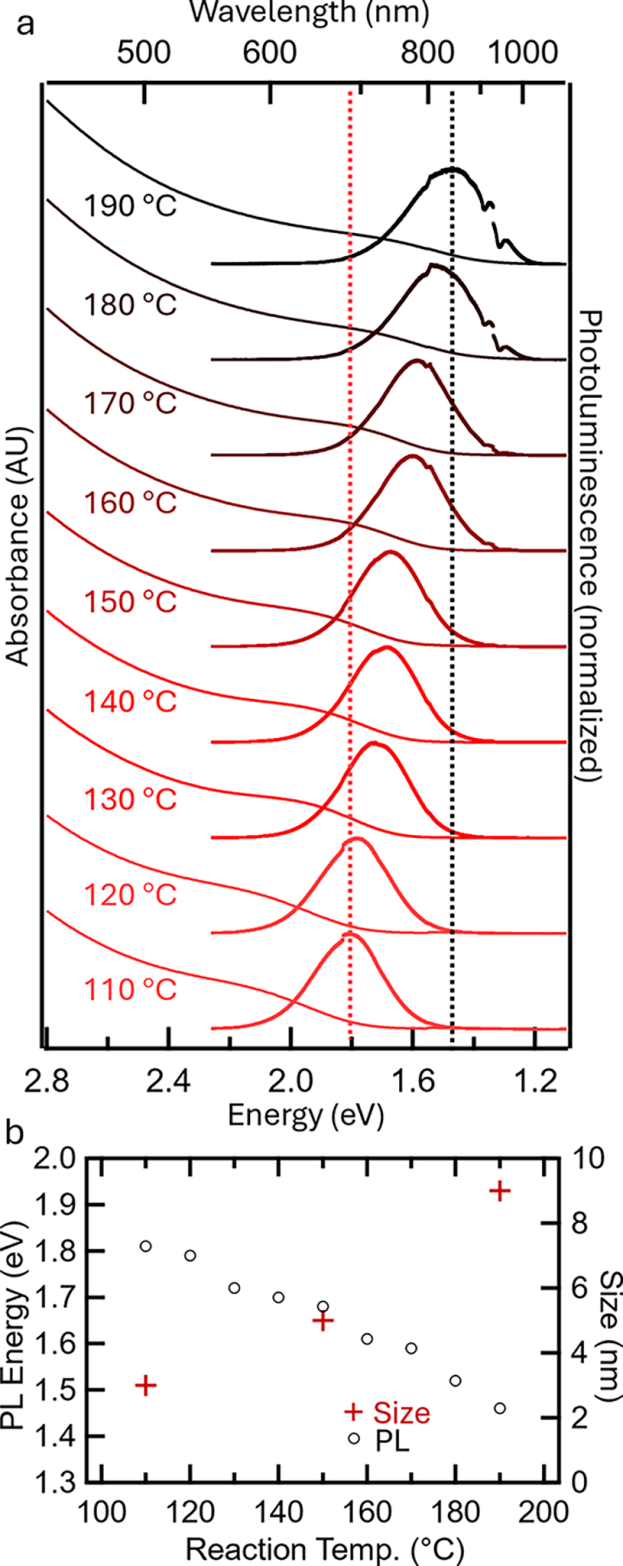
(a) Absorbance and PL (λ_ex_ = 405 nm; 3.06 eV) of BaCd_2_P_2_ QDs synthesized at various injection temperatures, as listed on the plot. The PL emission is tuned from 1.47 to 1.81 eV. The PL fwhm is 37 meV at 110 °C and 40 meV at 190 °C growth temperatures. The feature at 1.3 eV is due to a detector change. (b) Plot of the PL energy and QD size vs reaction temperature. Values are shown in Table S1.

The BaCd_2_P_2_ QDs were characterized by steady-state absorption and PL. Figure 1 shows the absorbance and PL spectra for QDs synthesized at 110–190 °C. The lower temperature synthesis produces smaller QDs, causing a blue shift in the absorbance and PL due to quantum confinement. In each case, there is a sloping onset of absorbance, which becomes sharper as the QDs decrease in size. An exciton absorbance peak is not clearly observed in these room temperature measurements, possibly due to the exciton binding energy being low in these Zintl-phase semiconductors. Polydispersity of the QD size is also a possible contributing factor to the sloping absorbance onset. The full width at half-maximum (fwhm) of the PL in BaCd_2_P_2_ QDs is 36–40 meV, which is broader than the bulk BaCd_2_P_2_ PL (10 meV),^1^ indicating that the PL broadening is due to polydispersity in these samples, a target to reduce in future synthesis refinement or by size selective precipitation techniques. At the highest temperature (190 °C), the absorption onset is approximately 1.43 eV and the PL position is 1.47 eV, in good agreement with the measurements on bulk BaCd_2_P_2_.^1^ Injection and growth at increased temperatures did not result in further red-shifting of the band edge (Figure S1 shows the growth at 220 °C).

**Figure 2:**
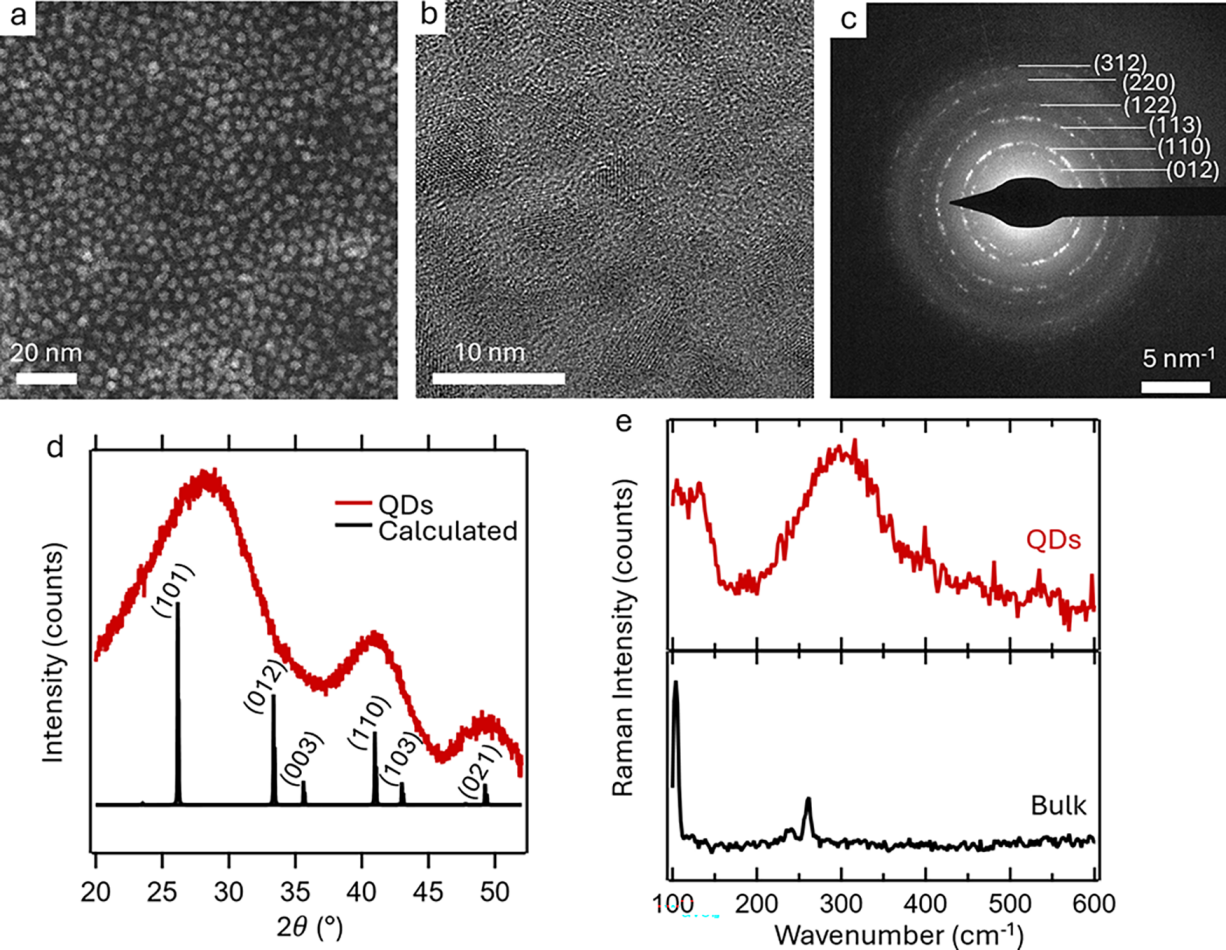
Images and phase characterization of BaCd_2_P_2_ QDs. (a) STEM HAADF of the BaCd_2_P_2_ QDs synthesized at 150 °C. (b) TEM images of larger BaCd_2_P_2_ QDs synthesized at 190 °C highlighting lattice fringes. (c) Selected area electron diffraction, which can be indexed to the *P*3̅*m*1 bulk phase. Values are shown in Table 1. (d) XRD of the BaCd_2_P_2_ QDs with a calculated bulk PXRD pattern for BaCd_2_P_2_ (space group *P*3̅*m*1). (e) Raman spectra of the QDs showing two discrete features in both bulk and the QDs.

High angle annular dark field (HAADF) scanning transmission electron microscopy (STEM) and transmission electron microscopy (TEM) images (Figure 2a,b and Figures S2,S3) show small particles (3–9 nm in size depending on the growth temperature) with no clear faceting or crystal habit. The particles can be loosely described as spherical. This lack of faceting is possibly due to agglomeration of small particles during growth or the trigonal symmetry of BaCd_2_P_2_ lacking robust surface energies for cubic or hexagonal facets (space group *P*3̅*m*1, no. 164).^16^ Transmission electron microscopy (TEM) on well washed particles showed lattice fringes, indicating crystallinity in these samples. The lattice fringe distance observed was 0.23 nm, which could correspond to the (110) reflection plane (Figure S2c). Selected area electron diffraction (SAED) collected on the sample in Figure 2c was much more conclusive and could be successfully indexed to the same structure as that of bulk BaCd_2_P_2_ (values in Table 1). Interestingly, before identifying adequate washing procedures to remove unreacted precursor, we were unable to observe lattice fringes or clear SAED patterns in the microscopy.

**Table 1:** Measured and Literature Values of Lattice Planes from SAED Patterns as Shown in Figure 2c

Measured (Å)	(*hkl*)	Calculated (Å)	Difference (Å)
2.65	(012)	2.68	0.03
2.26	(110)	2.20	0.06
1.62	(113)	1.66	0.04
1.36	(122)	1.35	0.01
1.13	(220)	1.10	0.03
1.03	(312)	1.02	0.01

To further confirm the crystal structure of produced BaCd_2_P_2_, the QDs were dispersed in chloroform and drop-cast onto Si wafer substrates for powder X-ray diffraction (XRD), Raman spectroscopy, and X-ray fluorescence (XRF). The XRD pattern (Figure 2d) on particles showed broad features that can be assigned to the (101), (110), and (021) diffraction reflections of the bulk *P*3̅*m*1 structure. The (101) reflection does appear to be shifted or broadened to higher 2θ angles, which indicates a possible element of strain contracting the lattice in these samples to be characterized in future experiments with a brighter X-ray source. Raman spectroscopy was compared between previously synthesized bulk BaCd_2_P_2_ powders and BaCd_2_P_2_ QDs (Figure 2e). Bulk BaCd_2_P_2_ has two sharp Raman peaks at 104 and 262 cm^–1^. The BaCd_2_P_2_ QDs grown at 190 °C showed two broadened peaks centered at 120 and 300 cm^–1^. This blue shift in the Raman peaks is expected with dimensionality confinement, and the presence of two peaks in both samples helps confirm that studied samples are Zintl-phase BaCd_2_P_2_ QDs. XRF was used to determine there was a 36:65 Ba:Cd ratio, close to the expected 1:2. This slight deviation could be due to Ba–P surface termination in these high surface area QD particles, yet further experiments will be required to determine this conclusively. The culmination of these results, as well as the SAED, allows us to conclude that the produced QDs are the BaCd_2_P_2_ Zintl-phase initially targeted.

## Optical Properties

**Figure 3:**
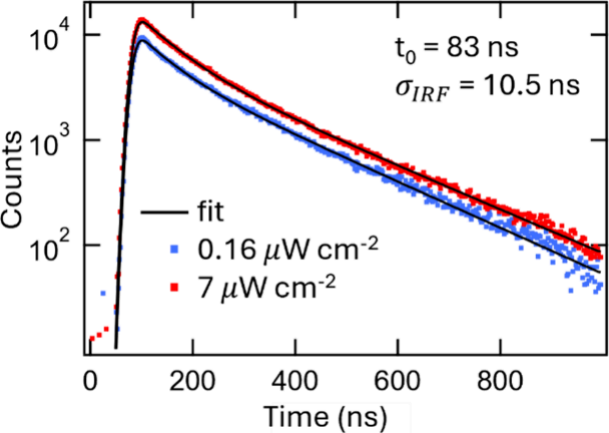
Time resolved photoluminescence (TRPL) of BaCd_2_P_2_ QDs (λ_ex_ = 500 nm; 2.48 eV). Plot of the PL decay with a biexponential fit produced decay lifetimes of 61 ns (25%) and 200 ns (75%) (power = 7.0 μW) and 70 ns (33%) and 210 ns (67%) (power = 0.16 μW).

Photoluminescence quantum yield (PLQY) was determined in a BaCd_2_P_2_ QD sample grown at 190 °C to be 21% (Figure S5). This is comparable with the initial demonstration of halide perovskite [(CH_3_NH_3_)PbBr_3_] colloidal nanocrystals (PLQY = ca. 20%)^17^ which was later improved with refined synthesis and purification methods to near unity.^18^ We further explored the carrier recombination lifetimes via time-resolved photoluminescence (TRPL) (Figure 3). Fitting the TRPL spectra to a biexponential decay convolved with a Gaussian instrument response function (σ_IRF_ = 10.5 ns) results in lifetimes of 61 ns (25% of integrated emission (IE)) and 200 ns (75% IE) (power = 7.0 μW) and 70 ns (33% IE) and 210 ns (67% IE) (power = 0.16 μW). The decay is distinctly biexponential but not distinctly power-dependent to suggest bimolecular recombination. Rather, the biexponential decay indicates that there are two populations decaying at their own independent rate. This could be due to polydispersity in the QD size or heterogeneity in the density of surface states, providing different overall decay rates for different QDs. This ∼160 ns weighted average lifetime is potentially suitable for a range of optoelectronic applications.

## Thin-Film Processing and Characterization

**Figure 4:**
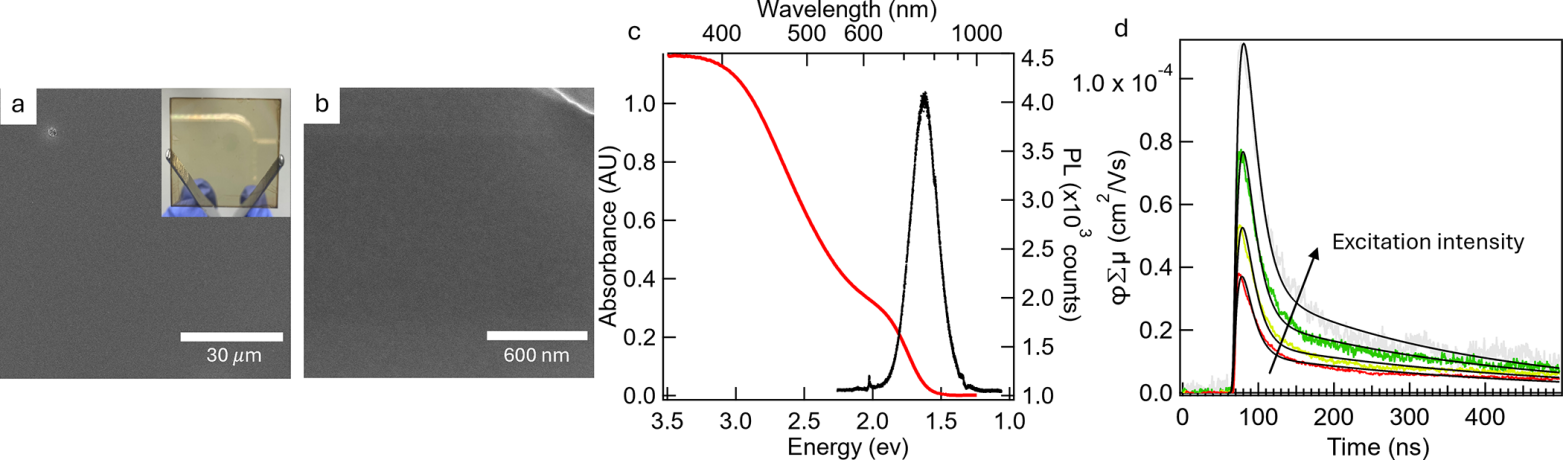
Thin films of BaCd_2_P_2_. (a,b) SEM images of the BaCd_2_P_2_ QD thin film surface with specks of dust in the corners to show focus. The inset shows an optical image of the thin film. (c) UV–vis and PL of the BaCd_2_P_2_ QD thin film. Further images are shown in Figures S6 and S7. (d) TRMC photoconductivity transients of the BaCd_2_P_2_ QD thin film with absorbed photon flux ranging from 2 × 10^14^ to 2 × 10^15^ cm^–2^, along with biexponential fits (black curves) of the data.

A key experiment in the development of novel semiconductors is the processing into thin films for potential integration into optoelectronic platforms. Thin films of QDs can be spin coated from solution, yet with native ligands, these films are challenging to grow into optically dense films (typically <0.1 optical density (OD)). As such, we utilized previously developed solid-state ligand exchange methods to remove long-chain ligands and replace them with small metal anion salts.^19,20^ This allows for repeated spin coating without removal of the underlying layer. In brief, BaCd_2_P_2_ QDs in octane were spin-cast onto glass slides followed by dipping into saturated cadmium(II) acetate (Cd(Ac)_2_) solutions as the ligand exchange salt. The thin films were characterized by scanning electron microscopy (SEM), which showed a smooth, pinhole free surface across multiple magnifications (Figure 4a,b and Figures S6,S7). Figure S6 shows cross sectional SEM of smooth films with ∼500 nm thickness corresponding to 8 repeated spin coating/ligand exchange cycles. Fourier transform infrared spectroscopy (FTIR) was performed on neat QDs (*i.e*. no ligand exchange) and the ligand-exchanged QD, both scraped off substrates for the measurement (Figure S8). FTIR shows the removal of long chain species and replacement of them with acetate, indicating the ligand exchange was successful. This provides a basic framework upon which to expand for BaCd_2_P_2_ QD film formation.

Time-resolved microwave conductivity (TRMC)^21,22^ was used to probe photoinduced charge generation and recombination dynamics within BaCd_2_P_2_ QD thin films prepared with an OD_500 nm_ > 0.6 by repeating the solid-state exchange procedure 8 times (Figure 4c). In most inorganic semiconductors, the yield of electron–hole pairs per absorbed photon (φ) can be assumed to be 1 in GHz-frequency TRMC measurements, making these quantitative assessments of the carrier mobility (diffusivity) on a 100 ps time scale.^23^Figure 4d shows photoconductivity transients and their biexponential fits over an order of magnitude excitation intensity. The average weighted lifetime (τ) is ca. 60 ns, with a longer time component of ∼300 ns corresponding to ca. 17% weight of the fit and an intensity-independent decay profile. The yield mobility (μ) product φΣμ_*t*=0_ is 2–4 × 10^–4^ cm^2^ V^–1^ s^–1^, which appears to be much smaller than that measured previously for powders of BaCd_2_P_2_.^1^ This result suggests that the TRMC signal originates primarily from intra-QD carriers (*i.e*. confined within a QD), which dramatically curtails the measured mobility. Calculations based on the size of the QD (5 nm) suggest that the intrinsic mobility of the material is 10 cm^2^ V^–1^ s^–1^ (analysis shown in Figure S9), explaining this apparent discrepancy between the bulk and QDs. Other novel semiconducting QD systems initially showed similar sub-100 ns lifetimes and mobilities in the 10^–4^ cm^2^ V^–1^ s^–1^ range and over time have been improved significantly via modifications to the synthesis, ligands, and processing.^23−25^ Cd(Ac)_2_ ligands are adequate for providing a pathway for thin-film formation, but other ligand removal (such as sintering) or exchange procedures are hypothesized to yield longer lifetimes and higher intergrain mobilities.

## Spontaneous Superlattice Formation and Cation Exchange Reactions

**Figure 5:**
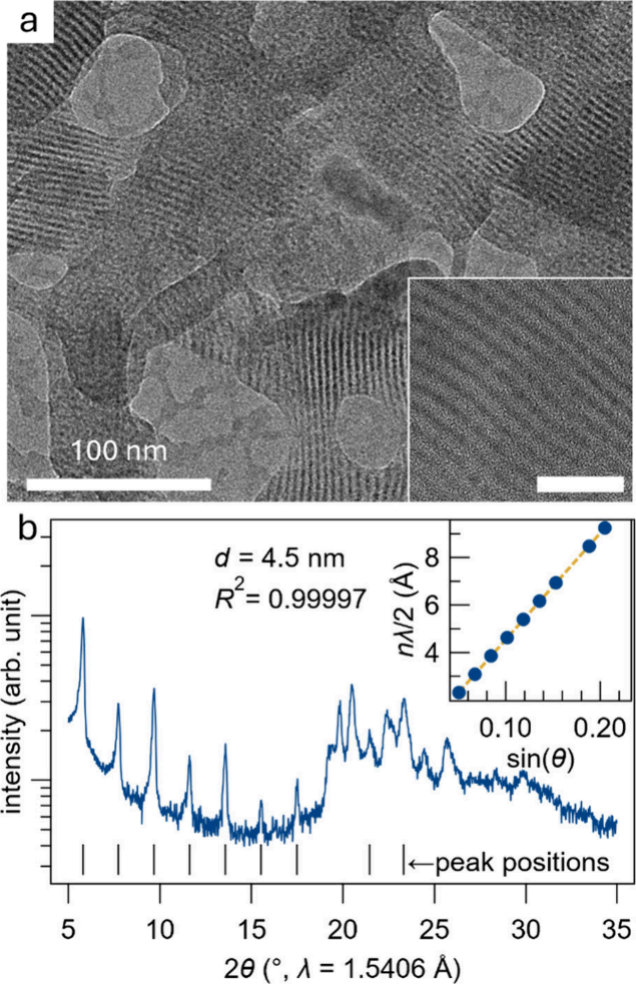
Self-assembly of the QDs into superlattice structures showing excellent packing. (a) TEM image of quantum dots self-assembled into a structure resembling a superlattice with a 4–5 nm spacing between higher contrast inorganic layers (the QDs). The inset shows a zoomed-in image (scale bar 20 nm). (b) XRD on a superlattice with a fit of 4.5 nm spacing, in excellent agreement with the TEM images. The inset is a linear fit to peak index vs positions such that the slope is *d*.

An interesting phenomenon is the self-assembly of these BaCd_2_P_2_ QDs into superlattice-like structures. Self-assembled QD superlattices open up opportunities for exotic phenomena such as inter QD optical coupling, superfluorescence, and enhanced thermoelectrics.^26−28^Figure 5a shows a TEM micrograph of a superlattice-like structure of the QDs stacked with ligand shell organics in between. Initially, the structure looked like nanowires or on-edge nanoplates, but closer inspection revealed individual particles that assembled in a linear array (Figure 5a inset). X-ray diffraction (Figure 5b) shows the prominence of this remarkably ordered array of BaCd_2_P_2_ QDs. A fit of the diffraction peaks reveals 4.5 nm spacing, in excellent agreement with electron micrographs. Some of the peaks in the 19–26° range, corresponding to *d-*spacings from ∼3.4 to 4.6 Å, drastically reduce the fit quality, presumably because they arise from internal QD and/or ligand spacings and so are not included in the superlattice fit. In very homogeneous colloidal quantum dots, self-assembly of particles upon drying is not uncommon.^29^ What is interesting about these self-assembled BaCd_2_P_2_ QDs is that the structure looks like a true superlattice, *i.e*. 2D stacked ordering instead of 3D arrays typically observed. Possibly, there is anisotropic ligand binding on the surface of these QDs driving this anisotropic formation.

**Figure 6:**
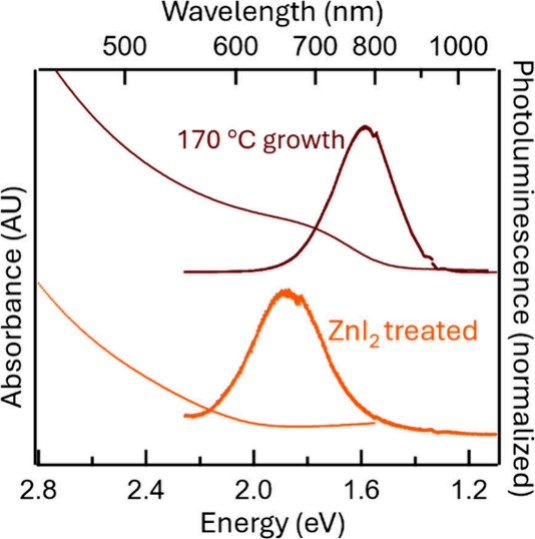
Cd/Zn cation-exchanged Ba(Cd_1–*x*_Zn_*x*_)_2_P_2_ QDs. The curve/sample for 170 °C growth is the same as in Figure 1 and is the starting position of the absorbance/PL. The PL of the ZnI_2_-treated BaCd_2_P_2_ QDs is at 1.88 eV, indicating partial Zn exchange to form Ba(Cd_1–*x*_Zn_*x*_)_2_P_2_.

Lastly, we explored the possibility of cation exchange reactions with BaCd_2_P_2_ QDs. Owing to the large surface-to-volume ratio in QDs, cation exchange reactions can be extremely rapid.^30^ This allows for optoelectronic property tuning in this system as well as reducing Cd content.^31,32^ Previously grown CaZn_2_P_2_ thin films showed a direct band gap of 1.95 eV,^3^ while the bulk BaCd_2_P_2_ band gap is 1.45 eV,^1^ suggesting the exchange of Zn for Cd will blue-shift the absorbance/PL spectra of BaCd_2_P_2_. Solid ZnI_2_ powders were added to BaCd_2_P_2_ QDs in toluene and stirred overnight to target Ba(Cd_1–*x*_Zn_*x*_)_2_P_2_. There was a noticeable change in the color of the solution, from black to red. Figure 6 shows the resulting shifting of the PL from an initial 1.60 to 1.88 eV. This is higher energy than our smallest particle emission wavelength (3 nm, 1.8 eV), providing strong evidence this is due to Zn exchange to form Ba(Cd_1–*x*_Zn_*x*_)_2_P_2_. To further confirm that Zn is incorporated into the lattice, electron diffraction patterns of cation exchanged Ba(Cd_1–*x*_Zn_*x*_)_2_P_2_ QDs were collected (Figure S10) and show two clear diffraction rings corresponding to the (113) and (012) diffraction planes at smaller *d*-spacing than the original BaCd_2_P_2_ (Table S2 shows tabular data and Table S3 shows calculated lattice parameters). Furthermore, energy dispersive X-ray spectroscopy (EDX) shows a significant Zn content (Figure S10) in the QDs.

In summary, we demonstrate the first solution phase growth of colloidal BaCd_2_P_2_ QDs with sizes ranging from 3 to 9 nm. The growth is achieved via a hot injection of (TMSi)_3_P into ligand-solubilized Ba and Cd precursors. A discussion of the growth conditions and observations is presented, including solubilities of precursors, reaction temperature, ligand chemistries, and stoichiometric ratios. The BaCd_2_P_2_ QDs grown are the same phase as bulk BaCd_2_P_2_ (*P*3̅*m*1) as determined by SAED, XRD, Raman spectroscopy, and XRF. Photocarriers are long-lived (∼160 ns weighted average), and the PLQY is 21% with no designed surface passivation, suggesting these are high quality optoelectronic materials. Preliminary thin films were demonstrated by a simple spin coating and ligand exchange procedure. TRMCs of thin films show low mobilities between inter-QD transport, common for novel QD thin films, with clear opportunities to be enhanced based on calculated high intradot mobilities. We also demonstrate future areas of interest by showing that BaCd_2_P_2_ QDs can be grown as thin films, form superlattices, as well as a Zn/Cd cation exchange reaction to demonstrate versatility. Future work will involve exploration of green precursors such as aminophosphine reagents to replace (TMSi)_3_P, improving the size dispersity, as well as exploration of further electronic properties.^33−35^ This growth demonstration should enable the exploration of a broader class of Zintl-phase colloidal nanomaterials, exciting for a wide range of optoelectronic applications and fundamental phenomena.

## Materials and Methods

### Materials

All chemicals were used without further purification. BaI_2_·2H_2_O (98%), CdO (99.99% trace metal basis), ZnI_2_ (≥98%), (TMSi)_3_P (95%), 1-octadecene (for synthesis, ≥ 91.0%), oleic acid (technical grade, 90%), trioctylphosphine oxide (ReagentPlus, 99%), olelyamine (technical grade, 70%), and methyl acetate (MeOAc, ReagentPlus, 99%) were purchased from Sigma-Aldrich. Toluene (ACS grade, 99.5%) was purchased from Oakwood Chemical. Acetone (ACS, 99.5%) was purchased from VWR.

### Safety Note on Using (TMSi)_3_P

(TMSi)_3_P is pyrophoric and evolves into toxic gas upon exposure to moisture. Any vials, syringes, syringe tips, pipet tips, or other materials that came into contact with the (TMSi)_3_P were quenched with bleach immediately after use. A flame-retardant lab coat and a face shield should be worn during this process. Bleach is highly effective at neutralizing unreacted (TMSi)_3_P. In addition, it should be noted that while not perfect, storing the (TMSi)_3_P for up to 1 week diluted in octadecene did not seem to affect the reactivity or growth reaction. Neat (TMSi)_3_P will smolder immediately upon exposure to air, whereas diluted (TMSi)_3_P does not visibly react upon air exposure. In either case, any materials in contact with diluted (TMSi)_3_P should be quenched with bleach immediately after use. In addition, when handling the QDs, “as synthesized” (*i.e*. formed particles in the growth mixture) bleach should be present nearby and signs of smoking should be watched for, in the case that unreacted (TMSi)_3_P is present from the growth and reacts upon exposure to air.

### Synthesis of BaCd_2_P_2_ QDs

A dried 25 mL three-neck round-bottom flask was charged with 73 mg (0.17 mmol) of BaI_2_·2H_2_O, 44 mg of CdO (0.34 mmol), 8 mL of ODE, 200 mg of TOPO, and 300 μL of OA. The mixture was dried on the Schlenk line (close to 100 mTorr) at RT until all bubbling stopped (<30 min) and then the temperature was raised to 115 °C and held for at least 1 h. Afterward, the atmosphere in the flask was switched to N_2_ and the mixture was heated to 220 °C and held for 30 min, ensuring complete precursor dissolution. The solution was then cooled to 120 °C and dried under vacuum again for at least 1 h. The solution was then transferred back to a nitrogen atmosphere and held at the desired temperature, and (TMSi)_3_P (98 μL, 0.34 mmol) dissolved in dry ODE (1:5 ratio) was swiftly injected and allowed to react for 30 min, followed by removal from heat and cooling to RT with no intentional quenching. For experiments done with OLAM, 0.5 mL of OLAM was used in the growth. Other deviations are noted in the text.

### Purification of BaCd_2_P_2_ QDs

After the growth was complete and the reaction mixture was cooled to RT, the mixture (typically ∼10 mL of mixture) was transferred to a 50 mL conical tube. MeOAc was added in a 1:1 ratio and centrifuged at 5K RPM for 5 min. The orange/brown supernatant was discarded, and the black precipitate was resuspended in 10 mL of toluene and then crashed out with 20 mL of acetone, followed by centrifuging at 10K RPM for 5 min. This acetone step was repeated, and the final precipitate was either resuspended in toluene or kept as a dry powder.

### UV–vis–NIR Steady State Spectroscopy

BaCd_2_P_2_ QDs dispersed in toluene were diluted, and UV–vis–NIR absorption spectra were collected with a Cary 6000i spectrometer.

### Photoluminescence (PL)

PL spectra were acquired by using a Horiba spectrophotometer equipped with a 405 nm laser with a collection time of 0.5 s and 600 line/mm grating.

### X-ray Fluorescence Spectroscopy (XRF)

Measurements were recorded using a Rh anode at 50 keV and spectra were modeled as a BaCd_2_P_2_ composition.

### Raman Spectroscopy

Raman spectra were recorded using a Renishaw inVia confocal system with a 532 nm laser line and a 20× long working distance objective lens equipped with an 1800 lines mm^–1^ grating.

### Time Resolved Photoluminescence (TRPL)

TRPL data were collected using a Hamamatsu Streak Camera (300–900 nm, C10910–04) with an NKT supercontinuum fiber laser (SuperK EXU-6-PP) routed to an acousto-optic tunable filter (SuperK Select). The samples were photoexcited at 500 nm with a rep rate of approximately 0.691 MHz and a spot size of a 2 mm diameter circle. Powers used are noted in the figure. The samples were loaded into a 1 cm cuvette and excited at right-angle geometry to the detector to minimize scattering.

### Electron Microscopy

Images were taken of QDs dispersed on ultrathin-lacey carbon grids. The grid was prepared by diluting the QDs in toluene until the color was barely visible and adding one drop via a Pasteur pipet onto the grid. The grid was then dried at 0.1 Torr overnight before being loaded into the electron microscopes. Most samples were briefly plasma cleaned in a mixture of 5% H_2_ + 95% N_2_ prior to imaging. The samples were examined in either a Thermo Fisher Spectra 200 cold field emission gun (FEG) Cs-corrected S/TEM or an FEI Tecnai F20 UltraTwin FEG STEM, both operated at 200 kV. Scanning electron microscopy images of the films were collected using a Hitachi S-4800 scanning electron microscope.

### X-ray Diffraction

Samples were prepared on a Si wafer for X-ray diffraction characterization by drop casting QDs dispersed in toluene. In samples that exhibited the superlattices, the sample was prepared from drop casting the QDs dispersed into chloroform. The presence of the ordered superlattice in electron microscopy from samples prepared from toluene suggests the chloroform is not essential. QD diffraction was collected on a Bruker D8 discover in grazing incidence geometry. Superlattice diffraction was collected on a Rigaku Smartlab using a surface aligned goniometer in locked-coupled geometry.

### Film Growth

Substrates (either quartz for TRMC or indium-doped zinc oxide (ITO) on glass for imaging) were prepared by sonicating for 15 min in isopropanol followed by 15 min UV-Ozone treatment. QD solutions were prepared utilizing the entire product of a washed reaction (based on 0.34 mmol Cd, same as above) grown at 160 °C with oleic acid and oleylamine ligand dissolved into 0.5 mL of octane. 35 μL of QD solution was deposited onto the cleaned substrates and spread evenly across the substrate with a pipet tip followed by spin coating for 30 s at 1500 rpm. The substrate was then dipped into a saturated Cd(Ac)_2_/MeOAc solution (0.22 g Cd(Ac)_2_ in 40 mL of MeOAc) for 5 s, followed by dipping into neat MeOAc for 5 s, followed by drying with clean N_2_ gas. This was repeated 8 times for the TRMC experiments, and between each cycle, an increase in thickness could be observed. Following the last spin coating reaching the desired thickness, the films were annealed at 100 °C to remove residual solvents.

### Fourier Transform Infrared Spectroscopy (FTIR)

FTIR was collected on a spin-coated thin film of ligand exchanged BaCd_2_P_2_ scraped off a substrate with a razor blade. For the control, a solution of washed BaCd_2_P_2_ QDs suspended in toluene was drop cast onto a silicon substrate and scraped off with a razor blade. Spectra were recorded with a Nicolet iS50 FTIR spectrometer.

### Cation Exchange Reaction

BaCd_2_P_2_ QDs suspended in toluene (1 mL ≈ 1/5 of reaction products based on 0.34 mmol Cd) were stirred overnight (∼18 h) with 0.32 g of ZnI_2_ added (ZnI_2_ is not fully solubilized). The reaction was centrifuged for 5 min at 6K RPM to remove ZnI_2_ powders followed by precipitation of the Ba(Cd_1–*x*_Zn_*x*_)_2_P_2_ QD supernatant with acetone and centrifuging 5 min at 10K RPM. The QD powders were diluted in toluene for further characterization.

### Photoluminescence Quantum Yield (PLQY)

The PL quantum yield measurements were conducted by using a custom-built spectrometer. Excitation light was provided by an EQ-99x Laser Driven Light Source (Energetique inc.) monochromated using a double-monochromator (Princeton Instruments HRS-500, 2×, 300 nm Blaze, 1200 g/mm gratings) which was fiber coupled into a 6 in. diameter LabSphere. Coupling optics images the fiber output onto a 1 cm cuvette with a machined Teflon cap mounted in the center of the sphere. All internal parts of the sphere are coated with Spectraflect, Spectralon, or Teflon. The total emitted + transmitted spectrum was measured through a fiber port with no direct line of sight to the sample cuvette to ensure that only scattered light was collected. Emission measurements were made using a Princeton Instruments HRS-300 spectrometer using an 800 nm blaze, 150 g/mm grating, coupled to a Princeton Instruments Pylon100F silicon CCD detector. All measurements were carried out with a 0.4 s exposure time and averaged over 50 collection frames, binning ∼50 rows of the 100 pixel high sensor.

### UV–vis of Films

Absorbance spectra of BaCd_2_P_2_ QD thin films prepared on quartz substrates were collected on a Cary 6000i UV–vis–NIR spectrophotometer. The samples were placed inside an integrating sphere and illuminated through a quartz substrate. Absorbance spectra were collected from 1000 to 300 nm with 1 nm resolution and a scan rate of 600 nm/min. For each sample, the fraction of light absorbed at 532 nm was extracted for TRMC analysis.

### TRMC

All TRMC measurements were carried out using a 532 nm excitation wavelength over at least an order of magnitude range of excitation power while continuously purging the microwave cavity with dry nitrogen. The 10 Hz excitation beam had a pulse width of approximately 5 ns, and the photoconductivity transients were recorded over a 500 ns time window. Biexponential fits of the TRMC transients include the pulsed nanosecond generation term as well as an instrument response function.

## Supporting Information Available

Additional figures giving high temperature growth to demonstrate the band gap position stops shifting at 190 °C growth temperature, electron microscopy images of different sized QDs and size distribution, XRF data, PLQY data, FTIR data, and further imaging of the films grown and a discussion of growth detailing solubilization of Ba precursors and exploring stoichiometry and ligand choice (PDF)
