# Supplementary material for: Synthesis and Characterization of Zintl-Phase BaCd2P2 Quantum Dots for Optoelectronic Applications
Source: ACS Nano. 2025 Mar 24;19(12):12345–53. doi: 10.1021/acsnano.5c02271 (PMC11966744; doi:10.1021/acsnano.5c02271)
Supplement: Supplementary file 1 — nn5c02271_si_001.pdf [file nn5c02271_si_001.pdf]

Supporting Information for Synthesis and Characterization of Zintl-Phase $BaCd_2P_2$ Quantum Dots for Optoelectronic Applications

Matthew P. Hautzinger,[1] Shaham Quadir,[1] Benjamin Feingold,[1,2] Reilly Seban,[1,3] Arianna J. Thornton,[1] Nikita S. Dutta,[1] Andrew G. Norman,[1] Ian A. Leahy,[1] Muhammad Rubaiat Hasan,[4] Kirill A. Kovnir,[4,5] Obadiah G. Reid,[1,6] Bryon W. Larson,[1] Joseph M. Luther,[1,6] Matthew C. Beard,[1,6] Sage R. Bauers[1*]*

[1]National Renewable Energy Laboratory, Golden, Colorado 80401, United States

[2] Department of Chemistry, University of Colorado, Boulder, Colorado 80401, United States

[3] Department of Physics, Colorado School of Mines, Golden, Colorado 80401, United States

[4]Department of Chemistry, Iowa State University, Ames, IA 50011, United States

[5]Ames National Laboratory, U.S. Department of Energy, Ames, IA 50011, United States

[6]Renewable and Sustainable Energy Institute, University of Colorado Boulder, Boulder, CO 80309, USA

Table of Contents

Section 1. Supplementary Figures and Tables 3
Figure S1. Absorbance and PL for $BaCd_2P_2$ QDs grown at 220°C. 3
Table S1. Reaction temperature and resulting properties. 3
Figure S2. Electron microscopy of $BaCd_2P_2$ QDs. 4
Figure S3. $BaCd_2P_2$ QD size distribution. 5
Figure S4. XRF of the $BaCd_2P_2$ QDs 5
Figure S5. PLQY of $BaCd_2P_2$ QDs grown at 190°C 6
Figure S7. Optical images of thin films. 8
Figure S8. FTIR of ligand exchanged thin-films. 9
Figure S9. Simulations of TRMC results. 9
Figure S10. TEM of $BaCd_2P_2$ Zn cation exchanged QDs 10
Table S2. Measured lattice distances in ZnI_2 exchanged $BaCd_2P_2$ QDs 10
Table S3. Calculated lattice constants from electron diffraction on ZnI_2 $BaCd_2P_2$ QDs. 10
Section 2. Notes about the growth 11
Figure S2-1. Stoichiometry and ligand impact on QD growth. 13
References 14

Section 1. Supplementary Figures and Tables

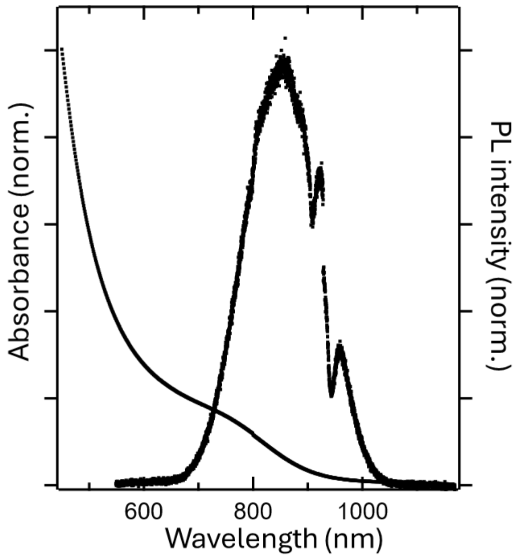

Figure S1. Absorbance and PL for $BaCd_2P_2$ QDs grown at 220°C. The photoluminescence (PL) maxima and abortion onset at 850 nm in agreement with bulk $BaCd_2P_2$ and the 190°C growth. Feature at ~950 nm is due to a detector change.

Table S1. Reaction temperature and resulting properties.

Temperature (°C)	Size (nm)	PL peak energy (eV)	FWHM (meV)
190	9	1.46	40
180	n/a	1.52	40
170	n/a	1.59	37
160	n/a	1.61	37
150	5	1.68	36
140	n/a	1.70	37
130	n/a	1.72	36
120	n/a	1.79	38
110	3	1.81	37

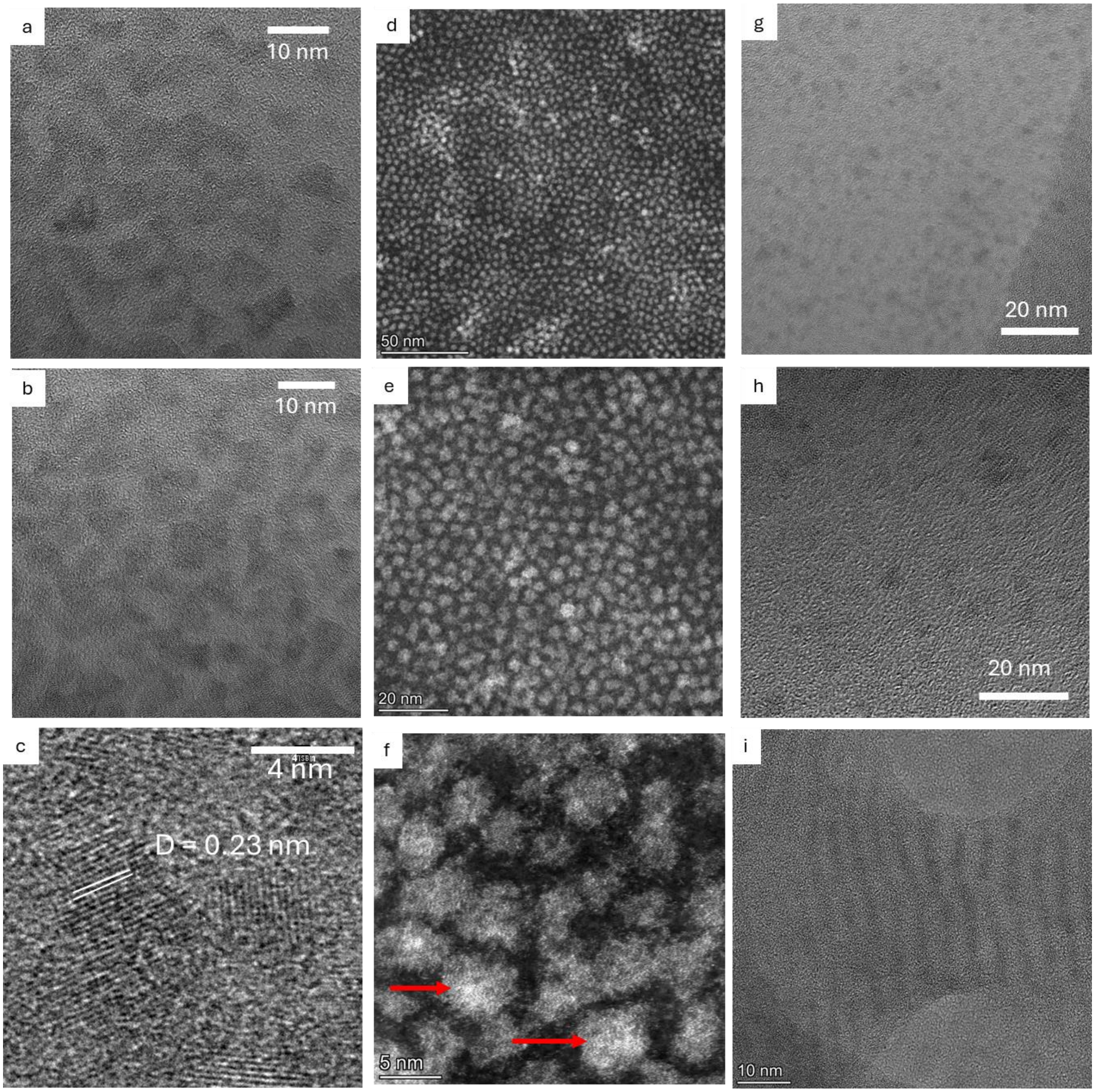

Figure S2. Electron microscopy of $BaCd_2P_2$ QDs. (a-c) TEM of particles grown at 190 °C. There is a wide range of shapes, possibly due to Ostwald ripening, but the particles are as long as 11 nm and as short as 7 nm (~9 nm). Panel c shows lattice distance of 0.23 nm in correspondence with the (110) plane. (d-f) STEM HAADF images of particles grown at 150°C with particles 5 nm in size. Panel f shows some areas of periodicity. We suspect the particles crystallinity is obscured due to washing procedures leading to excess Ba-ligand and Cd-ligand species present. (g-h) TEM of particles grown at 110°C with particles sized at 3 nm. (i) Packing of QDs into a superlattice-like structure.

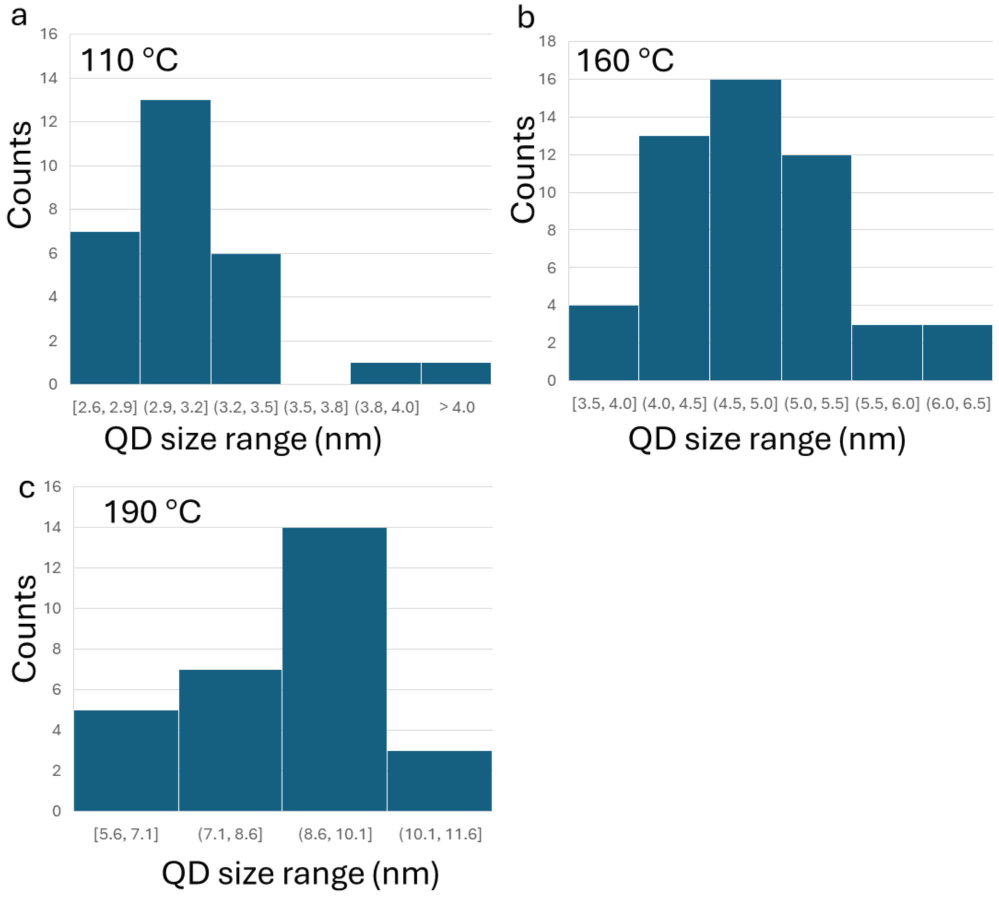

Figure S3. $BaCd_2P_2$ QD size distribution. (a) QDs grown at 110°C (~ 3 nm), (b) QDs grown at 150°C (~ 5 nm), and (c) QDs grown at 190°C (~ 9 nm).

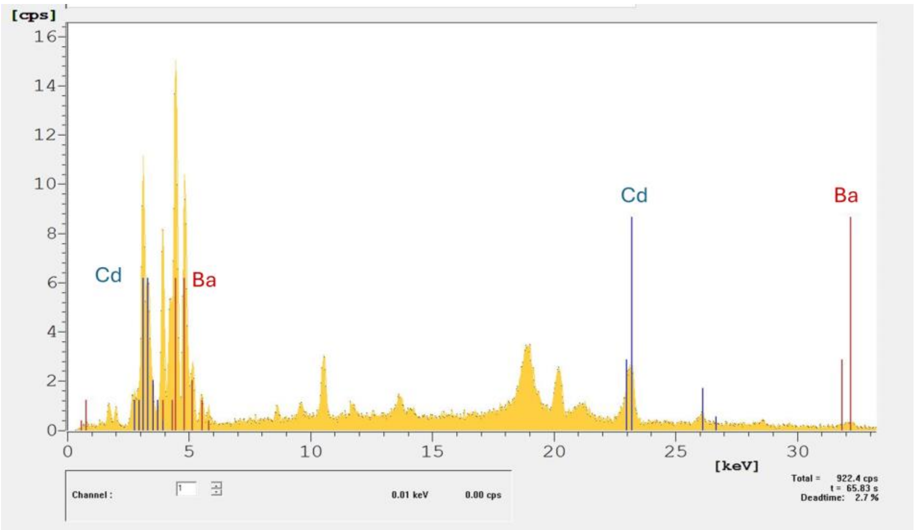

Figure S4. Raw XRF data of the $BaCd_2P_2$ QDs.

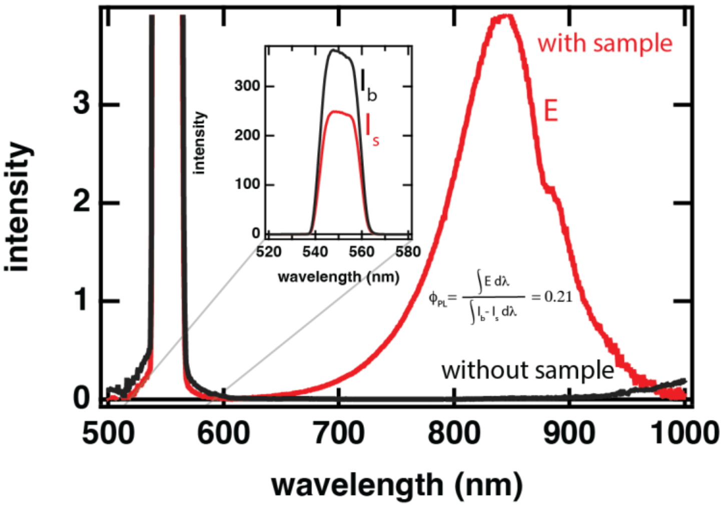

Figure S5. PLQY of $BaCd_2P_2$ QDs grown at 190°C. Total light output from an integrating sphere with (red) and without (black) the QDs in solution. The spectrum includes both transmitted excitation light and (where the sample is present) emitted light, with the inset showing a scaled representation of the excitation region. Excitation is centered at 550 nm with ~20 nm overall bandwidth. The emission band is evident between 650-1000 nm. The photoluminescence quantum yield is calculated by integrating the emission band and dividing it by the difference in integrated area in the excitation band, with the sample vs. pure toluene in the same cuvette. As shown in the inset, we find a value of 21%. Both spectra were background subtracted and had cosmic ray artifacts removed via linear interpolation. The small increasing tail in the toluene blank spectrum between 950-1000 nm is an imperfectly corrected artifact. Crucially for this measurement, these spectra are calibrated for relative spectral intensity using a calibrated quartz-tungsten halogen lamp (Princeton Instruments).

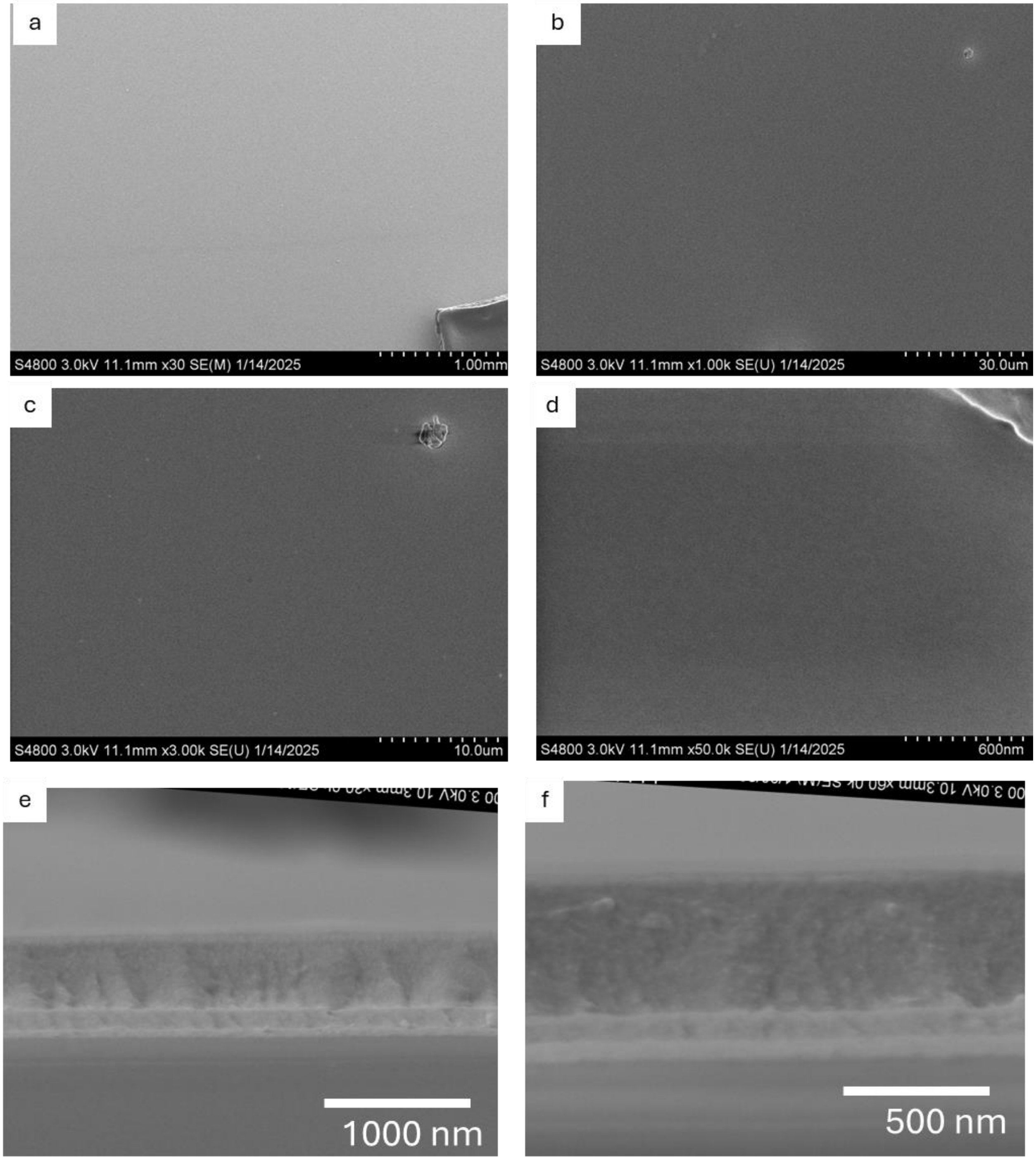

Figure S6. Additional SEM images of thin films. (a-d) SEM images of a $BaCd_2P_2$ thin film on ITO. Panels (b) and (d) are the full images of Figure 4. (e,f) Cross sectional SEM of $BaCd_2P_2$ QD thin film on ITO.

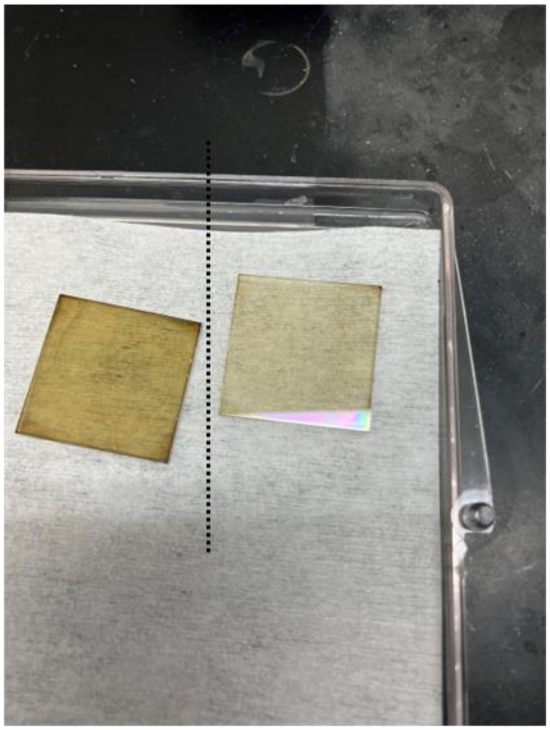

Figure S7. Optical images of thin films. Left side is a thin film coated with multiple solid-state ligand exchange repeats. Right side shows a single deposition with no ligand exchange procedure, highlighting the need for multiple depositions to build up a thickness.

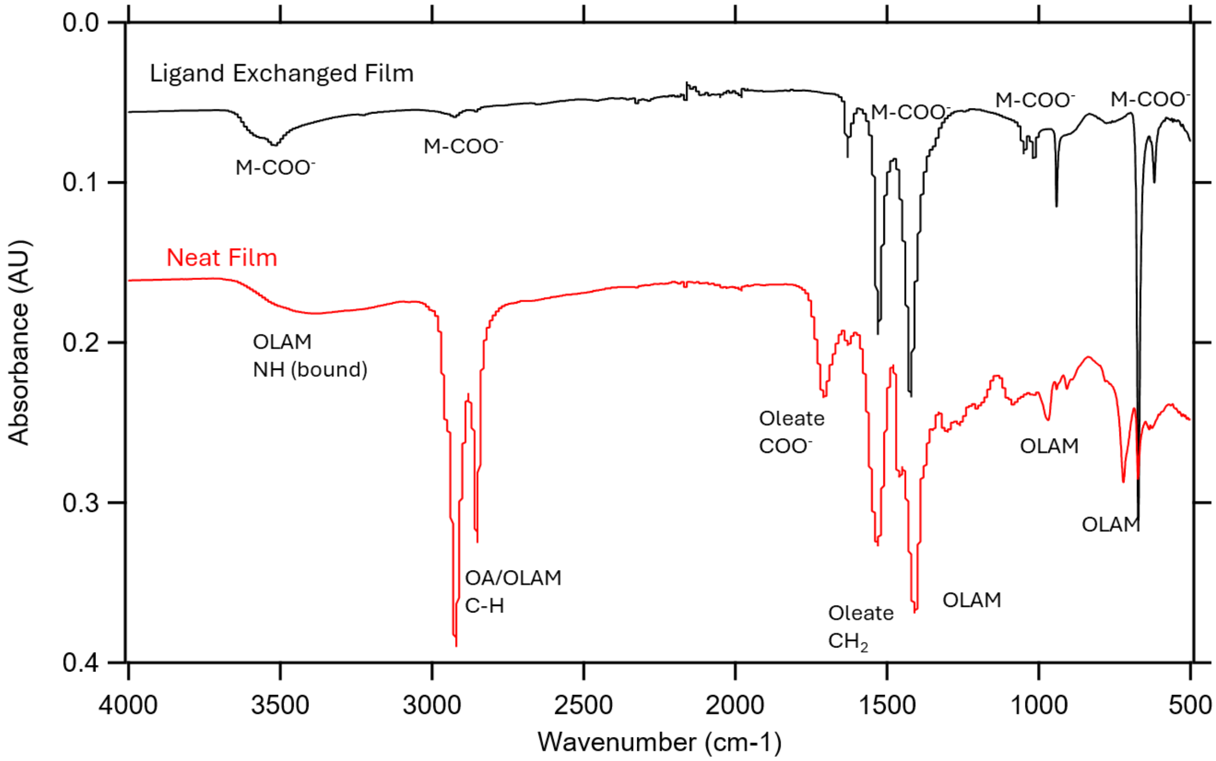

Figure S8. FTIR of ligand exchanged thin-films. The neat film is QDs spin coated and scraped off with no treatments and shows strong absorption at ~2900 cm^{-1} with two peaks indicative of long chain ligands (OA/OLAM). The peaks at 2900 cm^{-1} are not present in the ligand-exchanged films, yet two peaks around 1500 cm^{-1} persist corresponding to the metal-COO^- carboxylate vibrational modes present in metal acetates.

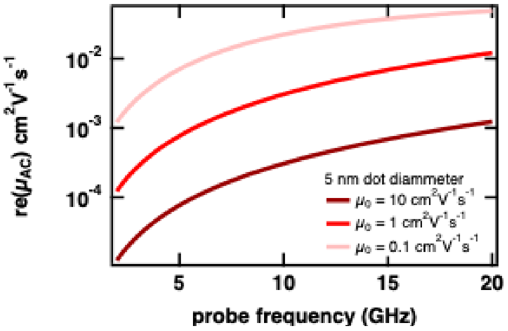

Figure S9. Simulations of TRMC results. Simulation results showing the real part of the complex mobility calculated using the Kubo formalism[1–3] describing the AC mobility of a charge carrier confined to a finite-sized object. The plot shows results for several different intrinsic mobilities from 0.1-10 $cm^2/V/s$ where the charge carrier is confined inside a 5 nm diameter quantum dot. The Y axis is the AC mobility one would expect to measure under these circumstances via TRMC, and the x-axis is the probe frequency employed. In our experiments, we use 9.8 GHz, indicating that our measured mobility (μ_{AC}) is consistent with an intrinsic mobility (μ_0) of ~ 10 $cm^2/V{\cdot}s$. i.e. if you grew a bulk sample of $BaCdP_2$ of the same quality

(scattering rate) the mobility would be 10 $cm^2/V{\cdot}s$. This is qualitatively consistent both with our prior measurement on a bulk powder of the same material, and theoretical calculations of same.[4]

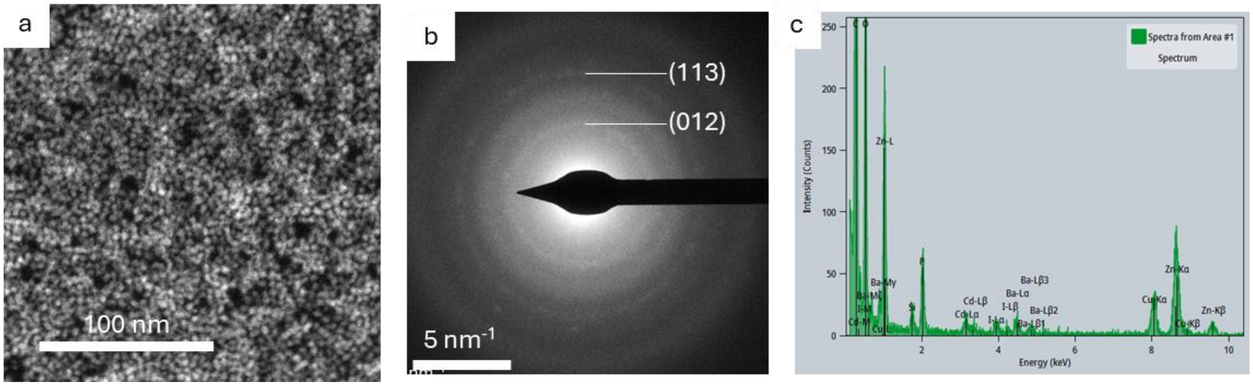

Figure S10. TEM of $BaCd_2P_2$ Zn cation exchanged QDs. (a) STEM-HAADF of the exchanged QDs. (b) Selected area electron diffraction pattern. (c) Energy dispersive x-ray spectroscopy of the exchanged QDs showing Zn incorporation (EDX).

Table S2. Measured lattice distances in ZnI_2 exchanged $BaCd_2P_2$ QDs.

Measured (Å)	Proposed (hkl)	$BaCd_2P_2$ Calculated (Å)	Difference (Å)
2.50	(012)	2.68	0.18
1.57	(113)	1.66	0.09

Table S3. Calculated lattice constants from electron diffraction on ZnI_2 $BaCd_2P_2$ QDs.

	Neat QD $BaCd_2P_2$	ZnI_2-treated QD $BaCd_2P_2$
a, Å	4.40	4.10
c, Å	7.55	7.30
V, Å^3	126.6	106.3

Section 2. Notes about the growth

$BaI_2 \cdot 2H_2O$ was the only Ba precursor we successfully dissolved in the OA/TOPO/ODE or OA/OLAM/ODE solution at 220°C (OA = oleic acid, TOPO = trioctylphosphine oxide, ODE = 1-octadecene, OLAM – oleylamine). We were unable to dissolve BaO, Ba(acetate)$_2$, and $BaCO_3$ under these conditions or even higher temperatures (up to 270°C). $BaI_2 \cdot 2H_2O$ would not dissolve with only OA present and required TOPO or OLAM to solubilize. Interestingly, our early experiments showed Ba(acetate)$_2$ can be solubilized by addition of NaI to the OA/TOPO/ODE precursor solution, possibly indicating the iodide is participating in coordination of the solubilized species, not simply a convenient barium salt for Ba-oleate formation. This was abandoned in favor of BaI_2 salts to avoid unintentional Na inclusion in the QDs. Based on the above observations we suggest a $BaI_2 \cdot x$OLAM species is what is formed as the QD precursor solution (as noted OLAM can be substituted for TOPO). Anhydrous BaI_2 as a starting material was also successfully solubilized with this preparation, suggesting water is not essential for solubilizing the Ba salt. However, the anhydrous Ba salt is less convenient for preparation of stoichiometrically accurate Ba concentrations when preparing our reactions in ambient atmosphere (as water may be picked up by the BaI_2 during weighing) and the hydrate was removed *in-situ* as discussed in the main text to seemingly great effect. We do suspect other halide salts may also be suitable, with potential to modify the precursor reactivity, but have not been attempted.

We explored a variety of growth conditions. First, we explored the effects of TOPO vs. OLAM as a ligand during growth. As can be seen in Figure S2-1a, there is no shifting in the PL spectra indicating similar growth dynamics and particle size. However, there is a much brighter PL emission intensity for the TOPO grown QDs. This may indicate either higher quality particles or better surface passivation with the TOPO ligand. We also modulated the phosphorous precursor concentration. The anion precursor during QD growth can control aspects of the growth such as nucleation. For example, in chalcogenide QD synthesis, the anion concentration and injection rate affects the size dispersity, yield, and amount of time before Ostwald ripening begins to occur in the growth.[5] In an experiment, we used "lean" anion injection of ratios 0.4, 0.7, and 1.0 (Cd/(TMSi)$_3$P molar ratio). Figure S2-1b shows there is blue shifting with decreasing (TMSi)$_3$P/Cd ratios, indicating smaller particles were grown. There is also an increase

in the PL width and the shape becomes asymmetric. Based on these observations, the highest quality $BaCd_2P_2$ QDs can be grown with TOPO and stoichiometric $(TMSi)_3P$.

The stoichiometric ratio of Ba:Cd during growth was not extensively explored. We generally find this pathway less interesting to explore at this stage, as Zintl-phases with different compositions were reported in Ba-Cd-P system: our target - $BaCd_2P_2$ (P-3$m1$),[6] Ba_2CdP_2 ($Cmc2_1$)[7], $Ba_2Cd_2P_3$ ($C2/m$),[8] and $Ba_3Cd_2P_4$ ($C2/m$).[8] With such a complex set of compounds we decided to keep the stoichiometric Ba:Cd ratio. Regardless, we did attempt 3:1 Ba:Cd precursor ratio for one growth and there appeared to be no formation of a visible/NIR absorbing compound, as indicated by the lack of color change after injection of the phosphorous precursor.

The ligand chemistry was lightly explored. As oleic acid is a protic ligand, excess oleic acid resulted in no reaction due to quenching of the $(TMSi)_3P$.[9] The other ligand species can have similar deleterious effects, either from purity or other side reactions. Attempts with large excess of olelyamine also resulted in no growth of particles for unclear reasons. Thus, OA was used only in slight excess molar ratio with CdO/BaI_2 and TOPO or OLAM were 1.5 molar ratio with $BaI_2 \cdot 2H_2O$; only enough excess to ensure solubilization of the precursors. Further exploration in this area may result in reduced polydispersity[10] which we find a valuable direction to pursue in future studies. Another note: the reaction seemed scalable and increasing the reaction molarity three-fold (while maintaining the ODE 8 mL volume) yielded the same growth as the lower concentration.

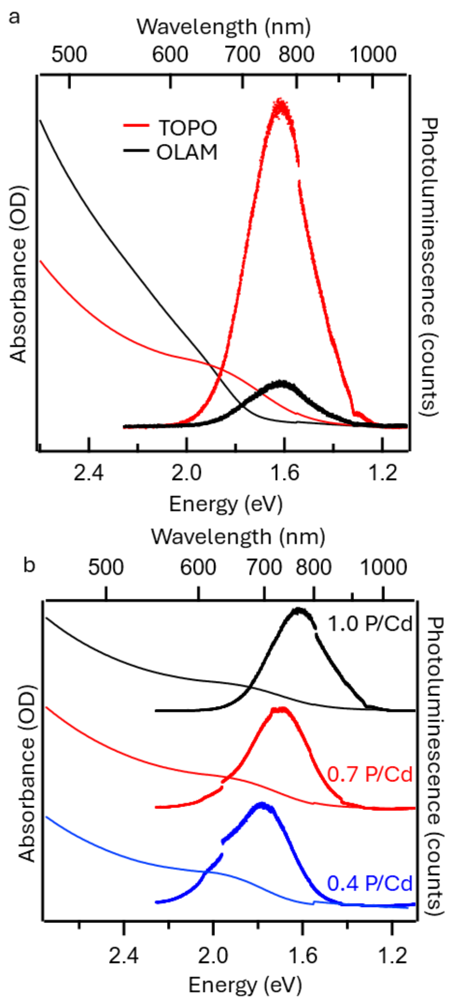

Figure S2-1. Stoichiometry and ligand impact on QD growth. (a) Comparison of TOPO vs. OLAM on the absorbance/PL in a 160°C growth temperature. For samples with similar concentrations, the TOPO growth produced much larger PL counts than OLAM. (b) Effects of $(TMSi)_3P$ lean conditions on absorbance/PL. Labeled on the plot is the ratio of $(TMSi)_3P$:CdO used during the growth.

References

(1) Bird, M. J.; Reid, O. G.; Cook, A. R.; Asaoka, S.; Shibano, Y.; Imahori, H.; Rumbles, G.; Miller, J. R. Mobility of Holes in Oligo- and Polyfluorenes of Defined Lengths. *J. Phys. Chem. C* **2014**, *118* (12), 6100–6109. https://doi.org/10.1021/jp5010874.

(2) Reid, O. G.; Yang, M.; Kopidakis, N.; Zhu, K.; Rumbles, G. Grain-Size-Limited Mobility in Methylammonium Lead Iodide Perovskite Thin Films. *ACS Energy Lett.* **2016**, *1* (3), 561–565. https://doi.org/10.1021/acsenergylett.6b00288.

(3) Prins, P.; Grozema, F. C.; Schins, J. M.; Siebbeles, L. D. A. Frequency Dependent Mobility of Charge Carriers along Polymer Chains with Finite Length. *Phys. Status Solidi B* **2006**, *243* (2), 382–386. https://doi.org/10.1002/pssb.200562719.

(4) Yuan, Z.; Dahliah, D.; Hasan, M. R.; Kassa, G.; Pike, A.; Quadir, S.; Claes, R.; Chandler, C.; Xiong, Y.; Kyveryga, V.; Yox, P.; Rignanese, G.-M.; Dabo, I.; Zakutayev, A.; Fenning, D. P.; Reid, O. G.; Bauers, S.; Liu, J.; Kovnir, K.; Hautier, G. Discovery of the Zintl-Phosphide BaCd2P2 as a Long Carrier Lifetime and Stable Solar Absorber. *Joule* **2024**, *8* (5), 1412–1429. https://doi.org/10.1016/j.joule.2024.02.017.

(5) Weidman, M. C.; Beck, M. E.; Hoffman, R. S.; Prins, F.; Tisdale, W. A. Monodisperse, Air-Stable PbS Nanocrystals via Precursor Stoichiometry Control. *ACS Nano* **2014**, *8* (6), 6363–6371. https://doi.org/10.1021/nn5018654.

(6) Klüfers, P.; Neumann, H.; Mewis, A.; Schuster, H.-U. AB2X2-Verbindungen Im CaAl2Si2-Typ, VIII [1] / AB2X2 Compounds with the CaAl2Si2 Structure, VIII [1]. *Z. Für Naturforschung B* **1980**, *35* (10), 1317–1318. https://doi.org/10.1515/znb-1980-1029.

(7) Balvanz, A.; Qu, J.; Baranets, S.; Ertekin, E.; Gorai, P.; Bobev, S. New N-Type Zintl Phases for Thermoelectrics: Discovery, Structural Characterization, and Band Engineering of the Compounds A2CdP2 (A = Sr, Ba, Eu). *Chem. Mater.* **2020**, *32* (24), 10697–10707. https://doi.org/10.1021/acs.chemmater.0c03960.

(8) Balvanz, A.; Baranets, S.; Bobev, S. Synthesis and Structural Characterization of the New Zintl Phases Ba3Cd2P4 and Ba2Cd2P3. Rare Example of Small Gap Semiconducting Behavior with Negative Thermopower within the Range 300 K–700 K. *J. Solid State Chem.* **2020**, *289*, 121476. https://doi.org/10.1016/j.jssc.2020.121476.

(9) Gary, D. C.; Cossairt, B. M. Role of Acid in Precursor Conversion During InP Quantum Dot Synthesis. *Chem. Mater.* **2013**, *25* (12), 2463–2469. https://doi.org/10.1021/cm401289j.

(10) Zhang, J.; Crisp, R. W.; Gao, J.; Kroupa, D. M.; Beard, M. C.; Luther, J. M. Synthetic Conditions for High-Accuracy Size Control of PbS Quantum Dots. *J. Phys. Chem. Lett.* **2015**, *6* (10), 1830–1833. https://doi.org/10.1021/acs.jpclett.5b00689.
